# Immunosensor Based on Antibody-Functionalized MoS_2_ for Rapid Detection of Avian Coronavirus on Cotton Thread

**DOI:** 10.1109/JSEN.2018.2829084

**Published:** 2018-04-23

**Authors:** Xuan Weng, Suresh Neethirajan

**Affiliations:** BioNano Laboratory, School of EngineeringUniversity of GuelphGuelphONN1G 2W1Canada

**Keywords:** Infectious bronchitis virus, molybdenum disulfide, FRET, immunosensor, cotton thread

## Abstract

Infectious bronchitis virus (IBV), an avian coronavirus, significantly affects the performance of both the egg-laying and meat-type birds causing the foremost of economic loss in poultry industry. This paper aims to develop a rapid, low-cost, and sensitive biosensor for IBV detection by using molybdenum disulfide (MoS_2_). MoS_2_ is a 2-D nanosheet which has strong high fluorescence-quenching ability when applied to a dye-labeled antibody (Ab). In this paper, we developed an Ab-functionalized MoS_2_-based fluorescent immunosensor, which utilized the fluorescence resonance energy transfer (FRET) between the MoS_2_ and fluorescence dye during the Ab–antigen interaction. The assay was performed on a low-cost cotton thread-based microfluidic platform due to the good wicking property and flexibility. Upon the optimization of assay conditions, the immunosensor demonstrated remarkable sensitivity of $4.6\times 10^{2}$ EID_50_ per mL and specificity with a dynamic linear response range of 10^2^–10^6^ EID_50_ per mL for IBV standard solutions. The developed immunoassay successfully detected the IBV spiked chicken serum with satisfactory results. The foregoing presents its potential application for on-farm detection.

## I. Introduction

Coronaviruses are enveloped, positive-strand RNA viruses of birds and mammals including humans. As a representative of the Gamma-coronavirus genus, the avian coronavirus (AvCoV) infectious bronchitis virus (IBV) may cause highly contagious respiratory disease in chickens and other galliforme birds [1]. IBV spread fast between individuals may cause a morbidity rate of 100% in bird populations which have not been vaccinated [2]. Chickens infected with IBV exhibit the symptoms of mild respiratory such as coughing, gasping, rales, and nasal discharge, and appearing depressed, or severe kidney and oviduct disease [3], [4], which may result in a decrease in egg production or quality. Development of affordable and highly sensitive detection methods for rapid monitoring and screening of IBV is extremely important, especially in less-developed countries. The enzyme-linked immunosorbent assay (ELISA) and polymerase chain reaction (PCR) test have now been used as gold standard for nucleic acid biomarker diagnostics. However, conventional ELISA can only work on a higher concentration of target molecules hence makes the approach not quite suitable for highly sensitive detection, while PCR usually requires expensive reagents and equipment as well as skilled personnel for the complicated process [5]. All these limitations restrict their applications in on-site detection.

Nanomaterials have become powerful element in constructing new biosensors due to their unique optical, electronic and catalytic properties [6]–[8]. Recently, transition metal dichalcogenides (TMDs) have gained world-wide attention as a group of 2D layered nanomaterials analogous to graphene owing to their excellent optoelectronics, nanoelectronics, and energy-harvesting properties. TMDs have been widely used in many research including DNA detection, transistors, photodetectors and photovoltaic devices [9], [10]. Molybdenum disulfide (MoS_2_) is an emerging material and one of such TMDs that can be synthesized in large scale and directly dispersed in aqueous solution and no treatment of surfactants or oxidation is required [11], [12]. Moreover, a higher fluorescence-quenching ability than graphene and the hydrophobicity property of surface make MoS_2_ become promising material in biosensing platforms and finding various applications [13]–[18]. The hydrophobicity of the MoS_2_ surface is a key enabling feature, because it enables strong affinity of protein-surface adsorption [11]. Kong *et al.* [11] made an aptamer-functionalized MoS_2_ biosensor by using the high fluorescence-quenching ability between MoS_2_ and dye-labeled single-stranded DNA probe for prostate specific antigen detection. High sensitivity and high selectivity with a detection limit for the PSA of 0.2 ng/mL were achieved. Tuteja *et al.* [18] reported a MoS_2_-based electrochemical immunosensor for detecting $\beta $-hydroxybutyrate, which is biomarker of subclinical ketosis. The immunosensor is based on the immmunodetection of the anti-$\beta $HBA antibodies immobilized on the MoS_2_-modified electrodes and $\beta $HBA antigen. Geldert *et al.* [19] developed fluorescence resonance energy transfer (FRET)-based MoS_2_ aptasensor for the detection of the malarial biomarker Plasmodium lactate dehydrogenase (pLDH). Zhang *et al.* [20] demonstrated a sandwich electrochemiluminescence immunosensor based on MoS_2_ for alpha fetal protein detection. MoS_2_ nanosheets surface was modified using polyethylenimine (PEI) polymer and gold nanopaticles. The immunosensor was able to analyze AFP in real human serum samples with limit of detection of 1.0 $\times \,\,10^{-5}$ ng/mL. These research demonstrate the huge protetial of MoS_2_ in biosensor applications.

The thread-based microfluidics is promising alternative to conventional microfluidic systems due to it many special characters. The thread-based biomedical devices are low cost and broadly available, flexible, easily to handle, lightweight, easy to manipulate and facilitate to transported or stored in any forms. In addition, the wicking properties of thread enable the low volumes of sample solution through it efficiently. All above mentioned makes thread-based microfluidics an attractive matrix for the fabrication of low-cost and low-volume microfluidic diagnostic devices for handheld on-site diagnosis applications [21]–[23].

**Fig. 1.**
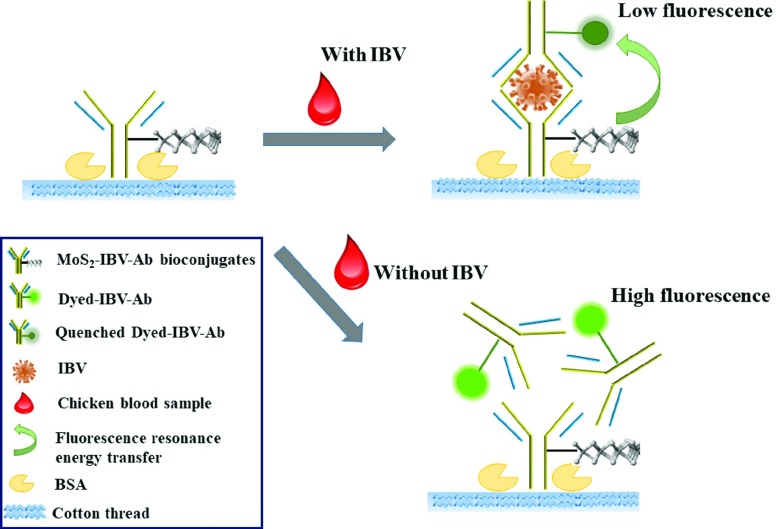
Schematic of the single-step homogeneous immunoassay on cotton thread using MoS_2_-based FRET for IBV detection.

Here, we aimed at developing a biosensor for rapid detection of IBV. We developed a single-step immunosensor on cotton thread based on antibody-functionalized MoS_2_ for the detection of IBV. The principle is based on a homogeneous FRET immunoassay, the sensing mechanism is shown in Fig. 1. The distinct quenching property of MoS_2_ on the fluorophore during the antigen-antibody reaction was adopted. The antibody (Ab) probes are modified with fluorescent dye labelling (dyed-IBV-Ab) and MoS_2_(MoS_2_-IBV-Ab bioconjugates), respectively. In the presence of IBV, both the dyed-IBV-Ab and the MoS_2_-IBV-Ab bioconjugates will specifically bind with the target IBV due to the antigen-antibody reaction. After binding, the fluorescence of the dyed-IBV-Ab probe is largely quenched owing to the transfer of electrons or energy between the closely connected dye molecules and the MoS_2_. In the absence of IBV, the fluorescence of the dyed-IBV-Ab probe will not change. Therefore, the concentration of the IBV in the chicken blood sample can be quantitatively determined by analysis the fluorescence intensity in an assay.

## II. Immunosensor Principle and Experiment

### A. Materials

MoS_2_ nano-sheet dispersion, ovine serum albumin (lyophilized powder, ≥96%), hydrogen peroxide (H_2_O_2_), poly(L-lysine) (PLL), chicken serum and all other mentioned chemicals and solvents were purchased from Sigma-Aldrich (Oakville, ON, Canada). Anti-infectious bronchitis virus (Massachusetts) (IBV) was purchased MyBioSource, Inc. (San Diego, CA, USA). Infectious bronchitis virus, low pathogenic avian influenza virus A H4N6 and H9N2 were kindly provided by our collaborator (Ontario Veterinary College, Canada) and the detailed culture procedure can be found elsewhere [24]. Briefly, isolate of IBV was propagated in embryonated specific pathogen free (SPF) chicken eggs followed by the titration determined by the method of Reed and Muench. The viral titer of the stock solution was $1\times 10^{6}$ EID_50_ per mL (egg infectious dose 50%). Avian influenza virus A H4N6 (Avian influenza virus A/Duck/Czech/56 (H4N6)) was propagated in 11-day-oldembryonated chicken eggs by inoculation into the allantoic cavity [25]. Inactivated Avian influenza virus A H9N2 (A/Turkey/Ontario/1/66) was propagated in 10-day-old embryonated SPF chicken eggs followed by inactivating with formalin (final concentration 0.02%) for 72 h at 37 °C [26]. Alexa Fluor 488 Antibody Labeling Kit was purchased from Life Technologies Inc. (Burlington, ON, Canada). Chicken whole blood (Cat. no. IR1-080N) was obtained from Innovation Research, Michigan, USA. Milli-Q water (18.2 $\text{M}\Omega$) was used in all experiments.

### B. Preparation of Dyed-IBV-Ab

The labelling of IBV Ab with fluorescent dye is conducted by using the Alexa Fluor 488 Antibody Labeling Kit. Briefly, 1.0 mg/mL IBV Ab was mixed with 1 M sodium bicarbonate solution. Then $100~\mu \text{L}$ of the mixture was added into the vial of Alexa FLuo dye followed by an incubation of 1 h at room temperature. The labeled antibody was then obtained via purification using a purification column with resin bed and centrifuged at $1100\times \text{g}$ for 5 min. The labeled antibody was stored at 4 °C for further use.

### C. Preparation of MoS_2_-IBV-Ab Bioconjugates

The MoS_2_ acted as a nanoplatform to adsorb IBV Ab and was further employed as a biological probe. The MoS_2_ (0.5 mg/mL) dispersion in aqueous was first concentrated by centrifugation at 8000 rpm for 30 min to remove the surfactant. The MoS_2_ was resuspended in Milli-Q water and sonicated for 1 h to provide a homogeneous solution. Aqueous poly(L-lysine) solution was added to the resulting MoS_2_ at a concentration of 1 mg/mL and stirred thoroughly for 1 h followed by overnight incubation at 4 °C. The poly(L-lysine) backbone is electrostatically adsorbed to MoS_2_ surface. The PLL-MoS_2_ mixture was purified and concentrated by centrifugation three times at 8000 rpm for 30 min to remove the solvent and resuspended in PBS. IBV Ab was then added at a desired concentration into the obtained mixture followed by overnight incubation at 4 °C to produce the MoS_2_-IBV-Ab bioconjugates. With the chemical modification by poly(L-lysine), the antibodies can be bind to MoS_2_ via electrostatic and covalent interactions [27]. The resulting mixture was purified at 5000 rmp at 4 °C and resuspended in PBS with desired concentrations for further use.

### D. Preparation of Thread-Based Microfluidics

**Fig. 2.**
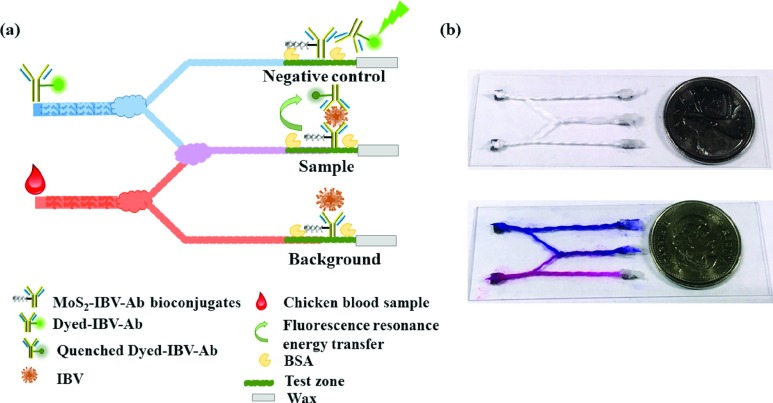
(a) Schematic of the thread network for immunosensor (not to scale). (b) A picture of the cotton thread microfluidics.

Cotton threads (100%) were boiled by 2 M NaCl for 30 min, and then soaked in 0.01% H_2_O_2_ and 0.01 M HCl for 5 min respectively, followed by washing with a large amount of ultrapure water to remove the residual acid. Finally, cotton threads were dried at 37 °C for at least 2 h and stored for further use. Cotton threads were rendered hydrophilic with an air plasma (Harrick Plasma, Ithaca, NY, USA). The design of the thread network is shown in Fig. 2. Two individual thread for sample and probe reagent dispensing were split to two streams. One of the two stream was then recombined with knot for mixing. Noted that the length of the split stream should be identical to achieve good mixing ratio between two streams flowing into it through the interwoven knot [21]. The other two streams are used for negative control (dyed-IBV-Ab solution, no IBV presented) and background testing, respectively. A test zones were defined on each branch thread. A test zone was coated with $3~\mu \text{L}$ (applied as three aliquots of $1~\mu \text{L}$, with 10 min drying at 37 °C after each step) of MoS_2_-IBV-Ab bioconjugates. The cotton thread was blocked with 1% BSA in PBS for 30 min, rinsed with PBS (0.1% Tween 20) and Milli-Q water, and quickly dried under a stream of N_2_. Paraffin wax was applied on the end of the each thread channels to create isolated zones to prevent the fluid flowing. The cotton thread was stretched on the glass slide with the paper support using double adhesive tape. The cotton threads with MoS_2_-IBV-Ab bioconjugates were stored at 4 °C and used within one week.

### E. Characterization

The morphology of MoS_2_ was characterized by the FEI-Tecani G2 transmission electron microscope (TEM) operating at 200 kV. The hydrodynamic diameters, size distribution and Zeta ($\xi$) potentials of the MoS_2_, PLL-MoS_2_ and MoS_2_-IBV-Ab bioconjugates were measured in water by a dynamic light scattering (DLS) system (Malvern Zetasizer Nano ZS, UK). The UV-Vis absorption spectra were analyzed on DR6000TM UV-Vis spectrophotometer (HACH, Loveland, Colorado, CA, USA) with a resolution of 1 nm. The fluorescence spectra were recorded by the Cytation 5 Multi-mode Reader (BioTek, Winooski, VT, USA). The fluorescent imaging (Ex/Em=488 nm/519 nm) was taken on a fluorescent microscopy (Nikon Eclipse Ti, Nikon Canada Inc., Mississauga, ON, Canada) under the same settings, namely exposure time, magnification, etc. The fluorescence intensity was then analyzed by analyzed by ImageJ.

### F. Operational Procedure

In a typical assay, a small volume ($30~\mu \text{L}$) of the fluorescence probe (dyed-IBV-Ab) and sample solution were added into the inlets of functionalized threads, respectively. An incubation time of 0~12 min at room temperature was then given to ensure the antigen-antibody reaction. After incubation, the fluorescence measurements were taken and recorded to analyze the change in fluorescence intensity using a fluorescence microscope equipped with a charge-coupled device camera. The intensity of the control zone and the background zone were used for calculation. Fluorescence images were converted into a numerical response using ImageJ software, a region of interest on the thread was drawn on the thread to perform the measurement.

### G. Validation and Optimization of Detection Conditions

The validation and optimization of the sensing mechanism was performed by fluorescent spectra analysis on a microplate reader. The effect of the concentrations of dyed-IBV-Ab ($10~\mu \text{g}$/mL, $20~\mu \text{g}$/mL and $30~\mu \text{g}$/mL), MoS_2_-IBV-Ab bioconjugates and incubation time on the fluorescence quenching were studied. Ten microliter of MoS_2_-IBV-Ab, sample and dyed-IBV-Ab were sequentially added in the microwell followed by fluorescence intensity analysis. All experiment was conducted in triplicate.

### H. Immunosensing of IBV in Chicken Whole Blood and Specificity

The performance of the presented immunosensor for detecting real blood sample was validated. IBV of various concentration (1, 5, $10\times 10^{4}$ EID_50_ per mL) was spiked in the chicken whole blood and loaded into the sensor. The average fluorescence intensity is then calculated to determine the sample concentration. To evaluate the specificity of the presented immunosensor, avian influenza A H4N6 and H9N2 were detected. The fluorescence signals with these non-specific analytes were recorded.

## III. Results and Discussion

### A. Characterization

**Fig. 3.**
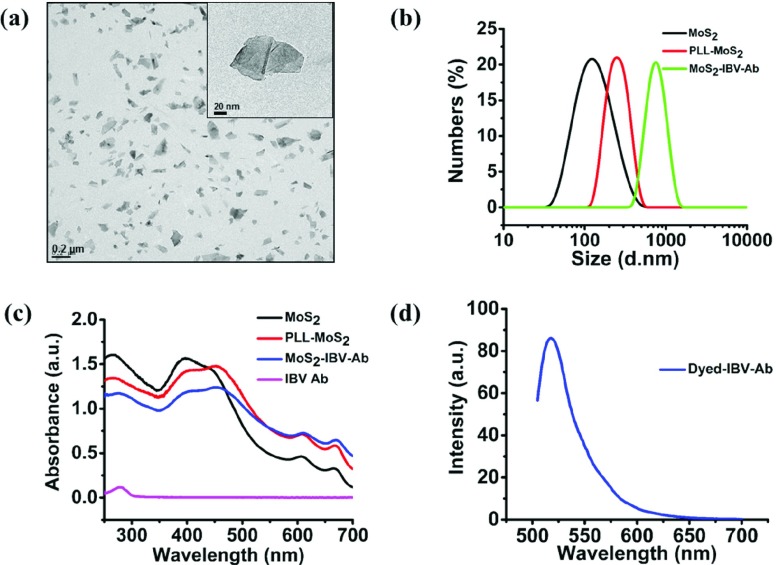
(a) TEM image of MoS_2_. (b) Particle size distribution of pure MoS_2_, PLL-MoS_2_, MoS_2_-IBV-Ab by DLS. The mean hydration diameter of the MoS_2_, PLL-MoS_2_, MoS_2_-IBV-Ab are 21.9 nm and 47.9 nm, respectively. (c) UV-Vis absorption spectrum for MoS_2_ dispersion in DI water, PLL-MoS_2_, MoS_2_-IBV-Ab and pure IBV Ab. (d) Fluorescence spectra of the dyed-IBV-Ab.

The structure and morphology of the MoS_2_ dispersion was investigated with the use of TEM (Fig. 3 (a)). TEM of the MoS_2_ nanosheets emphasized the uniformly sized nano MoS_2_ with a sheet-like morphology. The size distribution and zeta ($\xi$) potential of the MoS_2_, PLL-MoS_2_ dispersion and MoS_2_-IBV-Ab bioconjugates were characterized to evaluate the binding by dynamic light scattering (DLS) system (Malvern Zetasizer Nano ZS, UK). As shown in Fig. 3 (b), the size distribution of MoS_2_, PLL-MoS_2_ dispersion and MoS_2_-IBV-Ab bioconjugates are 113.5±0.32 nm, 162.1±1.07 nm and 983±84.5 nm, respectively. The Zeta potential ($\zeta$) values of MoS_2_, PLL-MoS_2_ dispersion and MoS_2_-IBV-Ab bioconjugates, as shown in Fig. S1, are −37.9±0.7 mV, +37.3±1.18 mV and −3.6±0.9 mV, respectively, which indicate the bind of the antibody and moderate dispersion of the antibody conjugated nanoparticles in aqueous medium [28]–[30]. The optical properties of MoS_2_ dispersion in DI water, PLL-MoS_2_, MoS_2_-IBV-Ab and pure IBV Ab was observed by UV-vis absorption measurement. As shown in Fig. 3(c), four characteristic peaks at ~398 nm, ~450 nm, ~612 nm and ~670 nm appeared which are due to band gap energies of 3.18 eV, 2.76 eV, 2.03 eV and 1.85 eV, respectively. The spectra is consistent with [31] and [32]. Fig. 3 (d) presents the fluorescence spectra of the dyed-IBV-Ab.

### B. Evaluation of Sensing Mechanism and Optimization

The validation and optimization of the biosensor was performed by a microplate reader. Standard IBV solution was made at a series of concentration of $1\times10$ (IBV1), $1\times 10^{2}$ (IBV2), $1\times 10^{3}$ (IBV3), $1\times 10^{4}$ (IBV4), $1\times 10^{5}$ (IBV5) and $1\times 10^{6}$ (IBV6) EID_50_ per mL.

**Fig. 4.**
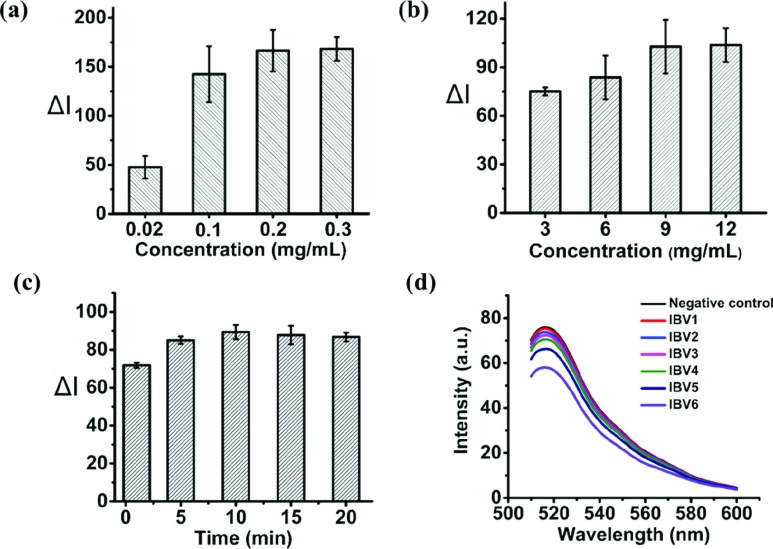
Sensing mechanism optimization and validation: (a) effect of concentrations of MoS_2_-IBV-Ab on the biosensor; (b) effect of concentrations of dyed-IBV-Ab ($3~\mu $ g/mL, $6~\mu \text{g}$/mL,$9~\mu \text{g}$/mL and $12~\mu \text{g}$/mL) on the biosensor; (c) effect of reaction time duration (1 min, 5 min, 10 min, 15 min and 20 min) on the biosensor; and (d) illustrative fluorescent spectra of the biosensor tested with multiple concentration of IBV standards ranging from 10~10^6^ EID_50_ per mL.

When preparing the thread, the MoS_2_-IBV-Ab conjugates was dropped on the thread, it filled up the micro-voids between thread fibers due to capillary action and got trapped between them. Additionally, upon drying, the conjugates also wrapped around the fibers. The effect of concentration of MoS_2_-IBV-Ab and dyed-IBV-Ab were investigated as well as the reaction time duration. A series of concentrations of MoS_2_-IBV Ab, 0.02 mg/mL, 0.1 mg/mL, 0.2 mg/mL and 0.3 mg/mL were carried out while keeping the concentration of dyed-IBV-Ab at $30~\mu \text{g}$/mL and the reaction time with 10 min. Fig. 4 (a) shows the relative fluorescence intensity difference ($\Delta \text{I}$) compared to negative control on the IBV standard of $1\times 10^{5}$ (IBV5) EID_50_ per mL. Result was taken at the moment of 10 min. A significant increase when the concentration of MoS_2_-IBV-Ab goes higher, however, no distinguishable difference is observed for the concentration higher than 0.2 mg/mL. Therefore, 0.2 mg/mL MoS_2_-IBV-Ab was selected as the optimized concentration. Similarly, the optimized concentration of the dyed-IBV-Ab was investigated by carried out the detection with a series of concentration, $3~\mu \text{g}$/mL, $6~\mu \text{g}$/mL, $9~\mu \text{g}$/mL and $12~\mu \text{g}$/mL. The result with a reaction time of 10 min is shown in Fig. 4 (b), the quenching effect increases as the increase of the concentration of the dyed-IBV-Ab while becoming “constant” when it goes above to $9~\mu \text{g}$/mL. The reaction time duration was also studied, an experiment was carried out by detecting the IBV standard of $1\times 10^{5}$ (IBV5) EID_50_ per mL using the optimized concentrations of MoS_2_-IBV-Ab and dyed-IBV-Ab, 0.2 mg/mL $9~\mu \text{g}$/mL, respectively. Time duration of 1 min, 5 min, 10 min and 20 min were investigated, the response of the sensing method is shown in Fig. 4 (c). The result shows that 10 min is more than sufficient to complete the quenching. Three triplicates were carried out for each data point and then the average of three independent measurements was calculated, the error bars indicate the standard deviation of the mean (n = 3). Under the optimized settings, fluorescence analysis for a series of IBV standard solution (from 0 to 10^6^ EID_50_ per mL, in PBS buffer of pH 7.4) was conducted, the result of which is shown in Fig. 4 (d). The result highlights a decrease in fluorescence intensity with the increased IBV concentration.

### C. Detection of IBV on Cotton Thread

**Fig. 5.**
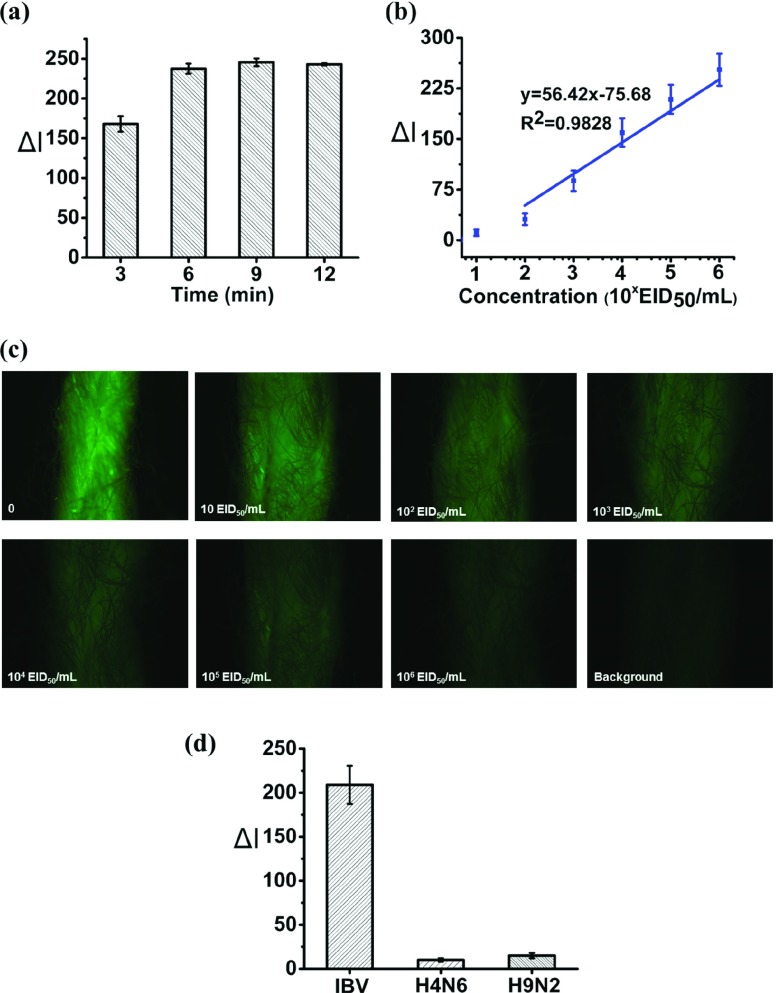
Detection of IBV on cotton thread. (a) Time effect on cotton thread under optimized settings. (b) Standard fluorescence intensity calibration plot against varying concentrations of IBV in standard PBS buffer (pH 7.4). (c) Representative fluorescence images of the test zones on the cotton thread upon the detection of various concentrations of IBV standard solution. (d) Specificity of the developed cotton biosensor with different influenza viruses. Error bars indicate the standard deviation of the mean ($n\,\,=3$).

With the optimization settings as demonstrated in previous section, quantitative analysis of IBV was carried out on the cotton thread. The transportation of fluidic was investigated firstly by using food color and it was observed that the fluid could reach the test zones within 1 min due to the capillary wicking. In addition, a time duration of 6 min was found to be sufficient for a complete reaction, as shown in Fig. 5 (a). The reaction time is slightly shorter that conducted in microplate. This may be attributed to the faster kinetics due to the better mixing effect on the thread. A wide range of IBV standard solution was detected. The IBV standard solution ranging from 0~10^6^ EID_50_ per mL was analyzed by the presented thread immunosensor to obtain the standard curve and calculate the limit of detection. A linear fit of the obtained relative fluorescence intensity difference with respect to varying concentration of IBV standards is plotted in Fig. 5 (b). The values denote average relative fluorescence intensity difference (n = 3) compared to negative control with standard deviation as error bars. It is shown that a distinct change presents starting from 10^2^ EID_50_ per mL with a correlation coefficient (R^2^) of 0.9828. The limit of detection calculated based on $3\sigma $ [33] of the blank is $4.6\times 10^{2}$ EID_50_ per mL. The different fluorescence responses of the test zones on the cotton thread upon the detection of various concentrations of IBV standard solution are shown in Fig. 5 (c). It is clearly seen that the fluorescence of the dyed-IBV-Ab was significantly quenched for all concentrations of IBV standard solution. With the concentration of IBV increases, the fluorescence is quenched more because more “sandwich” complex of dyed-IBV-Ab / IBV / MoS_2_-IBV-Ab are formed, resulting in largely quenched owing to the transfer of electrons or energy between the closely connected dye molecules and the MoS_2_.

### D. Accuracy of the Biosensor

The specificity of the developed cotton thread biosensor was evaluated against non-specific virus, avian influenza A H4N6 (100 HAU/$50~\mu \text{L}$) and H9N2 (100 HAU/$50~\mu \text{L}$). As shown in Fig. 5 (d), no distinguishable singles were obtained in the presence of the introduced interferents, which indicates that the highly specific towards to IBV.

ELISA was carried out side by side to validate the accuracy of the biosensor. Briefly, a ninety-six-well Maxisorp microtiter plate (Life Technologies Inc., Burlington, ON, Canada) was coated with the IBV Ab at 10 $\mu \text{g}$/mL diluted in filtered PBS (pH 7.4), and incubated overnight at 4 °C. The wells were washed with 0.05% Tween-20 ($1\times $PBS, pH 7.4) three times followed by being blocked with $200~\mu \text{L}$ of 3% BSA in PBS at room temperature for 2 h. After washing for three times, $100~\mu \text{L}$ of standards and spiked chicken blood were added, incubated for 1 h at room temperature and washed for three times with 0.05% Tween-20 ($1\times $PBS, pH 7.4). Into each well $50~\mu \text{L}$ of detection antibody dyed-IBV-Ab was added followed by incubation for 1 h at room temperature and washed with 0.05% Tween-20 ($1\times $PBS, pH 7.4) three times. $100~\mu \text{L}$ of filtered PBS (pH 7.4) was then added into each well and followed by the fluorescence intensity analysis (Ex=488 nm, Em=519 nm) on a microplate reader. All samples were tested in triplicate. Table I summarizes the results of the detection of IBV spiked chicken blood by presented immunosensor and ELISA. A good recoveries and consistency of the spiked IBV are presented. The standard derivations (SD) were 1.0~10% for both of methods. The total detection time from adding a sample was around 10 min. The results clearly demonstrated that the presented immunosensor is capable of the single-step detection of IBV in chicken blood sample and its high accuracy.

**TABLE I:** Comparison of Avian Detection Using Presented Immunosensor and ELISA Method

Spiked concentration ($\times 10^{4}$ EID_50_ per mL)	Immunosensor ($\times 10^{4}$ EID_50_ per mL)	SD (%)	ELISA ($\times 10^{4}$ EID_50_ per mL)	SD (%)
1.0	0.98	3.1	0.96	2.2
5.0	4.63	9.3	4.83	6.8
10.0	9.80	6.2	9.76	5.1
50.0	46.5	1.0	46.1	1.6

## IV. Conclusions

In this study, we reported a proof-of-concept MoS_2_-based immunosensor on a thread-based microfluidic network for rapid IBV detection. The distinct quenching property of MoS_2_ on the fluorophore during the antigen-antibody reaction was adopted. The thread-based network interconnected by knots to achieve the fluid mixing and separation. IBV standards and spiked chicken blood sample were successfully detection with high specificity and a detection of limit of $4.6\times 10^{2}$ EID_50_ per mL. The present immunosensor demonstrated a good linearity and validated with ELISA method. In comparison with conventional immunological tests, the presented method have many advantages, such as ease of local manufacture, small consumption of reagents and samples, high sensitive and short time of analysis. All these characteristics allow for the use of this technology for rapid, prompt on-site IBV detection.
